# Motivations for paediatric vaccine trial participation

**DOI:** 10.1186/s13063-023-07597-2

**Published:** 2023-09-08

**Authors:** Kushalinii Hillson, Jonathan Kantor, Marta Valente Pinto, Andrew J. Pollard, Dominic Kelly, Samantha Vanderslott, the AWARE clinical trial team

**Affiliations:** 1 Oxford Vaccine Group, Department of Paediatrics, University of Oxford, Oxford, UK; 2 Centre for Clinical Vaccinology and Tropical Medicine, NIHR Oxford Biomedical Research Centre, Churchill Hospital, Oxford, UK; 3 Oxford, UK; 4 Department of Engineering Science, University of Oxford, Oxford, UK; 5 Florida Center for Dermatology, St Augustine, USA; 6 Hospital Dona Estefânia, CHULC, Lisbon, Portugal

During the COVID-19 pandemic, clinical trials of vaccines received unprecedented publicity; whether this interest might be transferred to vaccine trials generally is unknown. Enrolment in paediatric COVID19 vaccine trials was slower than uptake of adult vaccine trials, and lessons learned are, therefore, of importance for future recruitment and participant experience. Previous studies have investigated motivations for participation in adult vaccine trials [1, 2], paediatric trials for chronic conditions [3], and select paediatric vaccine trials [4]. By contrast, for a non-COVID-19 paediatric vaccine trial, with recruitment from March–May 2021, we noted a higher rate of response (5·71%) than we have seen previously in the same population. The study was a randomised controlled trial of acellular vs whole cell pertussis vaccines (AWARE, part of the Periscope consortium). 295 responses were received; 184 respondents volunteered to participate, and 112 infants met the inclusion/exclusion criteria. For our study on trial motivations, 110 surveys were sent out and 81 responded (73·6% response rate). Baseline characteristics of the trial participants’ parents are outlined in  Supplementary Table 1.

Previously, we have observed response rates of 2%-4% for similar trials. We therefore hypothesised that prior vaccine trial experience and exposure might modulate the threshold for trial participation, and descriptively studied motivations and barriers to paediatric vaccine trial participation in this context. Ethical approval and feedback from the Oxford Vaccine Centre Public and Patient Involvement (PPI) group was received. Motivations for trial participation were dichotomised by prior trial participation (Table 1); self-described altruistic motivations were common, while motivations related to concrete personal benefits, regardless of prior trial participation, were less frequently reported. The two most cited motivations were improving the health of children and contributing to scientific progress, while access to in-home study visits was reported as a motivator by 83% of respondents. The pandemic context may have contributed to both the emphasis on scientific progress and the sense of public service and interest in trials, while also heightening the perceived benefit of in-home visits.

**Table 1:** Motivations for trial participation. *P*-values are for two-sample t-tests, without a correction for multiple comparisons

	**Overall (** ***n*** ** = 81)**	**Prior Trial Experience (** ***n*** ** = 16)**	**No Prior Trial Experience (** ***n*** ** = 65)**	***P*** ** value**
**Direct benefits to child**				
Vaccine access—protective	28 (34·6%)	4 (25·0%)	24 (36·9%)	0·38
Paediatrician access	39 (48·15%)	6 (37·5%)	33 (51·6%)	0·35
Immunisations at home	67 (82·7%)	10 (62·5%)	57 (87·7%)	0·02
GP appointments difficult	7 (8·64%)	1 (6·25%)	6 (9·23%)	0·71
Safer to receive vaccines at home	33 (40·74%)	5 (31·25%)	28 (43·08%)	0·39
**Altruism/ societal benefits**				
Improve health of children	79 (97·53%)	16 (100%)	63 (96·92%)	0·48
Scientific progress	80 (98·77%)	16 (100%)	64 (98·46%)	0·62
Others view positively	21 (25·93%)	6 (37·50%)	15 (23·08%)	0·24
Friends/ family positive reaction	57 (70·37%)	10 (62·50%)	47 (72·31%)	0·45
**Clinical trial experience**				
Wanted trial experience	40 (49·38%)	7 (43·75%)	33 (50·77%)	0·62
COVID changed my view	23 (28·40%)	6 (37·50%)	17 (26·15%)	0·37
**Pertussis-Specific Concerns**				
Comforted: vaccine previously approved in UK	77 (95·06%)	16 (100%)	61 (93·85%)	0·31
Comforted: vaccine approved by WHO	78 (96·30%)	16 (100%)	62 (95·38%)	0·39
Side effects worth improved efficacy	79 (97·53%)	15 (93·75%)	64 (98·46%)	0·28
Worried re severe reaction	14 (7·28%)	2 (12·50%)	12 (18·46%)	0·58
Worried about long term health effects	1 (1·23%)	0 (0%)	1 (1·54%)	0·62

This survey has limitations, being an unvalidated survey instrument, the reliance on self-report, and the inability to ascertain motivations among those who declined to participate. Further research including representative sample of the general UK population and better controlling for social desirability bias may shed further light on the nature and magnitude of differences in motivation, providing a basis for targeting adjustments to enrolment—and improve generalisability especially post-pandemic. Parental motivations for enrolling children in clinical trials are understudied and merit detailed exploration to maximise successful recruitment in future trials.

### Supplementary Information

**Additional file 1: Supplementary Table 1.** Baseline characteristics of 81 respondents. *P*-values are for chi-squared tests.

## References

[1] Detoc M, Launay O, Dualé C (2019). Barriers and motivations for participation in preventive vaccine clinical trials: Experience of 5 clinical research sites. Vaccine.

[2] Detoc M, Gagneux-Brunon A, Lucht F, Botelho-Nevers E (2017). Barriers and motivations to volunteers' participation in preventive vaccine trials: a systematic review. Expert Rev Vaccines.

[3] Brooks SP, Bubela T (2020). Application of protection motivation theory to clinical trial enrolment for pediatric chronic conditions. BMC Pediatr.

[4] Chantler TEA, Lees A, Moxon ER, Mant D, Pollard AJ, Fiztpatrick R (2007). The role familiarity with science and medicine plays in parents’ decision making about enrolling a child in vaccine research. Qual Health Res.

