# Supplementary material for: Motivations for paediatric vaccine trial participation
Source: Trials. 2023 Sep 8;24:574. doi: 10.1186/s13063-023-07597-2 (PMC10485999; doi:10.1186/s13063-023-07597-2)
Supplement: Supplementary file 1 — Additional file 1: Supplementary Table 1. Baseline characteristics of 81 respondents. P-values are for chi-squared tests. [file 13063_2023_7597_MOESM1_ESM.docx]

**SUPPLEMENTARY MATERIAL**

|  | **Overall (n=81)** | **Prior Trial Experience (n=16)** | **No Prior Trial Experience (n=64)** | **P** |
| --- | --- | --- | --- | --- |
| **Age range** |  |  |  | 0.62 |
| <25 | 2 (2·5%) | 0 | 2 (3·12%) |  |
| 25-34 | 43 (53·75%) | 10 (62·5%) | 33 (51·56%) |  |
| 35-44 | 35 (43·75%) | 6 (37·5%) | 29 (45·31%) |  |
|  |  |  |  |  |
| **Sex** |  |  |  | 1.0 |
| Female | 75 (93·75%) | 15 (93·75%) | 60 (93·75%) |  |
| Male | 5 (6·25%) | 1 (6·25%) | 4 (6·25%) |  |
|  |  |  |  |  |
| **Nationality** |  |  |  | 0.20 |
| British | 69 (85·2%) | 12 (75·0%) | 56 (87·50%) |  |
| Other | 12 (14·8%) | 4 (25·0%) | 8 (12·50%) |  |
|  |  |  |  |  |
| **Ethnic Origin** |  |  |  | 0.17 |
| Asian | 1 (1·23%) | 1 (6·25%) | 0 |  |
| Black | 2 (2·47%) | 0 | 2 (3·12%) |  |
| Mixed | 2 (2·47%%) | 0 | 2 (3·12%) |  |
| White | 75 (92·60%) | 15 (93·75%) | 60 (93·75%) |  |
|  |  |  |  |  |
| **Education** |  |  |  | 0.24 |
| GCSE | 1 (1·25%) | 0 | 1 (1·56%) |  |
| A-Levels | 5 (6·25%) | 0 | 5 (7·81%) |  |
| Bachelors | 24 (30·0%) | 3 (18·75%) | 21 (32·81%) |  |
| Postgraduate | 39 (48·75%) | 12 (75·0%) | 27 (42·19%) |  |
| Other Professional | 9 (11·25%) | 1 (6·25%) | 8 (12·50%) |  |

Supplementary Table 1. Baseline characteristics of 81 respondents. P-values are for chi-squared tests
